# Retroperitoneal Leiomyosarcoma Associated with an Elevated β-HCG Serum Level Mimicking Extragonadal Germ Cell Tumor

**DOI:** 10.1080/13577140020025904

**Published:** 2000-12

**Authors:** Michael Froehner, Hans-Juergen Gaertner, Andreas Manseck, Sven Oehlschlaeger, Manfred P. Wirth

**Affiliations:** 1 Department of Urology University Clinics ‘Carl Gustav Carus’ Technical University of Dresden Fetscherstrasse 74 Dresden D-01307 Germany; 2 Department of Pathology University Clinics ‘Carl Gustav Carus’ Technical University of Dresden Dresden Germany

## Abstract

*Patient.*  A 65-year-old man was admitted with a large primary retroperitoneal tumor and an increased β-human chorionic gonadotropin (β-HCG) serum level. A germ cell tumor was suspected; however, a computed tomography-guided biopsy failed to enable tumor classification. After two courses of chemotherapy, the β-HCG serum level had returned to the normal level and a diagnostic laparotomy with incisional biopsy was performed. The immunohistochemical examination of the specimen identified the tumor as a retroperitoneal pleomorphic leiomyosarcoma.

*Discussion.* Tumor markers play only a marginal role in the work-up of patients with soft tissue sarcomas. In men with suspected retroperitoneal sarcomas, however, the determination of germ cell tumor markers occasionally enables a preoperative distinguishing of primary retroperitoneal germ cell tumors with considerable consequences for management. In this setting, a retroperitoneal tumor associated with a moderately elevated β-HCG is a diagnostic dilemma, and surgeons should be aware of the pitfall of a β-HCG-producing leiomyosarcoma in the differential diagnosis.


*Sarcoma* (2000) **4**, 179–181

CASE REPORT

### Retroperitoneal leiomyosarcoma associated with an elevated β-HCG serum level mimicking extragonadal germ cell tumor

MICHAEL FROEHNER,[1] HANS-JUERGEN GAERTNER,[2] ANDREAS MANSECK,[1] SVEN OEHLSCHLAEGER[1] & MANFRED P. WIRTH[1]

[1]*Department of Urology and* [2]*Department of Pathology, University Clinics 'Carl Gustav Carus', Technical University of Dresden, Dresden, Germany*

**Abstract**

*Patient.* A 65-year-old man was admitted with a large primary retroperitoneal tumor and an increased β-human chorionic gonadotropin (β-HCG) serum level. A germ cell tumor was suspected; however, a computed tomography-guided biopsy failed to enable tumor classification. After two courses of chemotherapy, the β-HCG serum level had returned to the normal level and a diagnostic laparotomy with incisional biopsy was performed. The immunohistochemical examination of the specimen identified the tumor as a retroperitoneal pleomorphic leiomyosarcoma.

*Discussion.* Tumor markers play only a marginal role in the work-up of patients with soft tissue sarcomas. In men with suspected retroperitoneal sarcomas, however, the determination of germ cell tumor markers occasionally enables a preoperative distinguishing of primary retroperitoneal germ cell tumors with considerable consequences for management. In this setting, a retroperitoneal tumor associated with a moderately elevated β-HCG is a diagnostic dilemma, and surgeons should be aware of the pitfall of a β-HCG-producing leiomyosarcoma in the differential diagnosis.

**Key words**: *sarcoma, leiomyosarcoma, retroperitoneal, β-human chorionic gonadotropin, germ cell tumor, extragonadal*

### Introduction

Leiomyosarcomas producing β-human chorionic gonadotropin (β-HCG) are exceedingly rare with only three cases reported in the available literature, one each of spermatic cord,[1] small[2] and large intestinal[3] origin. We report, to our knowledge, the first case of a large primary retroperitoneal leiomyosarcoma associated with an elevated β-HCG serum level causing considerable difficulties in differential diagnosis.

### Case history

A 65-year-old man presented with a left-sided upper retroperitoneal tumor measuring $14 \times 16 \times 13$ cm$^3$ (Fig. 1). The β-HCG serum level was 48 U/l (Abbott AxSym Total β-HCG assay; Abbott, Wiesbaden, Germany; normal level <5 U/l), but α-fetoprotein (AFP) and placental alkaline phosphatase (PLAP) were within normal range. There was no evidence of a primary testicular tumor. A computed tomography (CT)-guided biopsy (Fig. 1) yielded no material suitable for histopathological classification. Because the location of the tumor and the elevated β-HCG serum level suggested an extragonadal germ cell tumor, an immediate onset of chemotherapy seemed to offer the best chance of cure. To repeat the CT-guided biopsy or to perform an explorative laparotomy was feared to cause further dangerous loss of time. Two courses of chemotherapy containing *cis*-platinum, etoposide and ifosfamide were applied. As was to be expected in a putative germ cell tumor, the β-HCG

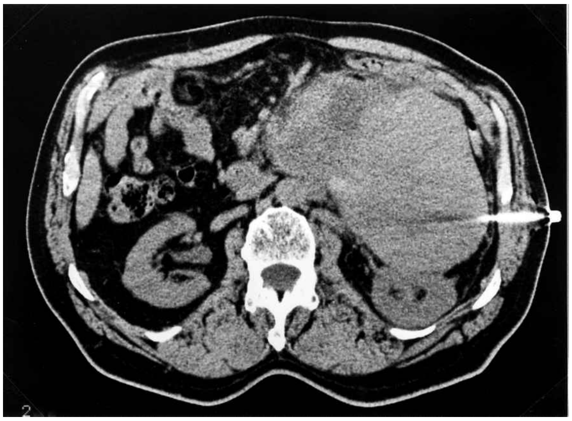

**Fig. 1.** *Partially necrotic retroperitoneal leiomyosarcoma at computed tomography-guided biopsy.*

Correspondence to: Michael Froehner, MD, Department of Urology, University Clinics 'Carl Gustav Carus', Technical University of Dresden, Fetscherstrasse 74, D-01307 Dresden, Germany. Tel. +49 351 458-2447; Fax. +49 351 458-4333; E-mail: wirth-m@rcs.urz.tu-dresden.de

1357-714X print/1369-1643 online/00/040179-03 © 2000 Taylor & Francis Ltd
*DOI: 10.1080/13577140020025904*

serum level decreased to 3.3 U/l after the first course and to 0.5 U/l after the second. Nevertheless, the tumor did not decrease in size and appeared uncommonly firm in physical examination. At diagnostic laparotomy, the tumor was found to be unresectable because of infiltration of the superior mesenteric vessels. An incisional biopsy was performed. The histopathological examination of the obtained material revealed a malignant mesenchymal tumor. Immunohistochemical staining was positive for desmin and smooth muscle actin (Fig. 2) and negative for epithelial markers (MNF116, Cam 5.2), germ cell markers (PLAP, β-HCG; Fig. 2A), and S-100 protein, classifying the neoplasm as pleomorphic leiomyosarcoma. Thirty-five days postoperatively, the β-HCG level has risen again to 26.1 U/l. The patient died of progressive disease 2 months after the laparotomy despite further chemotherapy.

### Discussion

Tumor markers play only a marginal role in the work-up of patients with soft tissue sarcomas. Male patients with suspected retroperitoneal sarcomas are an exception. Because of the difficult surgical accessability of these neoplasms, the danger of tumor cell spillage during biopsy and the problems of a histopathological classification based on small tumor samples, the germ cell tumor markers β-HCG, AFP and PLAP are occasionally the only available tool to distinguish the prognostically much more favorable germ cell tumors from retroperitoneal sarcomas before—in germ cell tumors, unnecessary—multivisceral *en bloc* resection. In this setting, a retroperitoneal tumor associated with a moderately elevated β-HCG level is a diagnostic dilemma and, as illustrated by the current case, the pitfall of a β-HCG-producing leiomyosarcoma should be taken into account.

Was the elevated β-HCG level in the current case caused by the described neoplasm? Gailani *et al.*[3] measured the β-HCG level in the plasma of 320 patients with non-trophoblastic neoplasms and 70 healthy controls. Only one of the healthy controls had an (insignificantly) increased β-HCG level. Of the 320 patients with non-trophoblastic neoplasms, 54 had an elevated β-HCG level; however, only three of them (0.9%) had β-HCG levels higher than a fourfold increase of the normal level, all three suffering from advanced metastatic disease. These data strongly support, together with the decrease of the β-HCG level during chemotherapy and the re-increase after the withdrawal of treatment, a relationship between the current neoplasm and the tumor marker despite the lacking demonstrability of β-HCG expression (perhaps an effect of chemotherapy) in the investigated tumor sample.

Although there are only few documented cases, it may be hypothesized that an elevated β-HCG level in leiomyosarcoma is an ominous prognostic sign. β-HCG-positivity has been found restricted to high-grade areas.[1,2] All available cases showed a distinctly unfavorable course with metastatic disease[1–3] and, in the current case, rapid progression. It is conceivable that an elevated β-HCG level in leiomyosarcoma indicates a tumor with a significant portion of fast-growing high-grade cells.

Luteinizing hormone/HCG receptors were found in human smooth muscle cells of various origin,[4,5] and the growth of human myometrial smooth muscle cells has been shown to be stimulated by HCG, possibly in a cAMP/protein kinase A signaling pathway.[6] Considering these data, it seems worth investigating whether β-HCG expression in leiomyosarcoma is only an epiphenomenon due to pathologic gene activation in highly degenerated cells[1] or whether it even stimulates tumor cells in an autocrine or paracrine pathway.

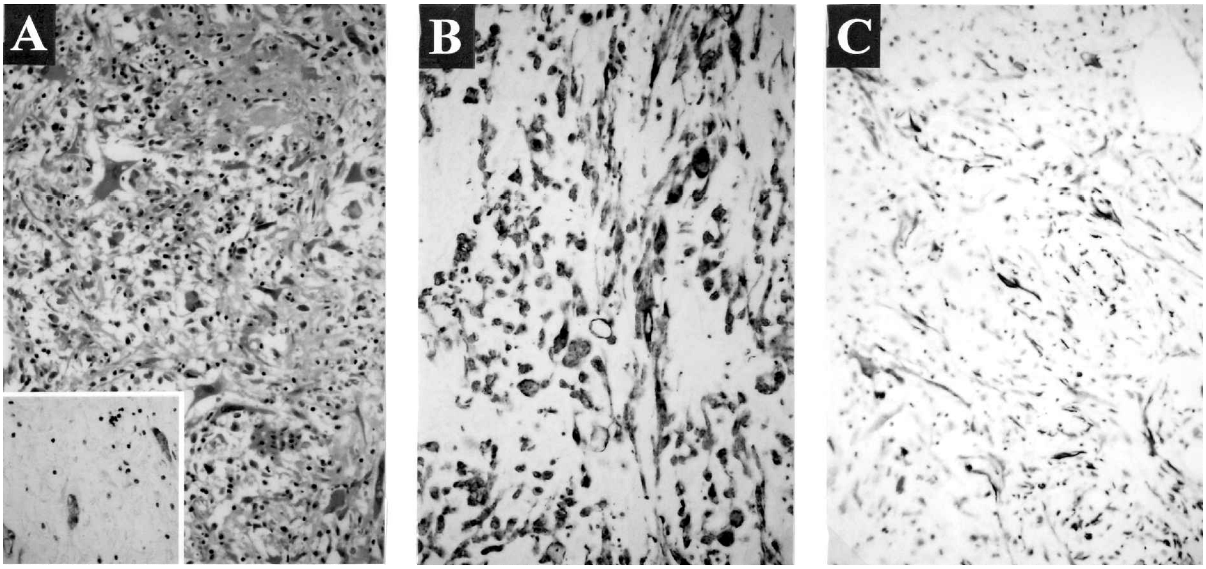

**Fig. 2.** *(A) Pleomorphic leiomyosarcoma (× 180, hemolysin and eosin) (inset: negative immunohistochemical staining for β-human chorionic gonadotropin), with positive immunohistochemical staining for (B) desmin and (C) smooth muscle actin. 1 Retroperitoneal leiomyosarcoma*

### References

1 Seidl C, Lippert C, Grouls V, Jellinghaus W. Leiomyosarkom des samenstrangs mit paraneoplastischer β-HCG produktion. *Pathologe* 1998; 19:146–50.

2 Meredith RF, Wagman LD, Piper JA, Mills AS, Neifeld JP. Beta-chain human chorionic gonadotropin-producing leiomyosarcoma of the small intestine. *Cancer* 1986; 58:131–35.

3 Gailani S, Chu TM, Nussbaum A, Ostrander M, Christoff N. Human chorionic gonadotropins (hCG) in nontrophoblastic neoplasms. Assessment of abnormalities of hCG and CEA in bronchogenic and digestive neoplasms. *Cancer* 1976; 38:1684–6.

4 Tao YX, Bao S, Ackermann DM, Lei ZM, Rao CV. Expression of luteinizing hormone/human chorionic gonadotropin receptor gene in benign prostatic hyperplasia and in prostate carcinoma in humans. *Biol Reprod* 1997; 56:67–72.

5 Tao YX, Heit M, Lei ZM, Rao CV. The urinary bladder of a woman is a novel site of luteinizing hormone-human chorionic gonadotropin receptor gene expression. *Am J Obstet Gynecol* 1998; 179:1026–32.

6 Kornyei JL, Lei ZM, Rao CV. Human myometrial smooth muscle cells are novel targets of direct regulation by chorionic gonadotropin. *Biol Reprod* 1993; 49:1149–57.
